# Smart nanogels for cancer treatment from the perspective of functional groups

**DOI:** 10.3389/fbioe.2023.1329311

**Published:** 2024-01-10

**Authors:** Jiachen Yu, Yuting Liu, Yingchun Zhang, Rong Ran, Zixiao Kong, Duoyi Zhao, Minda Liu, Wei Zhao, Yan Cui, Yingqi Hua, Lianbo Gao, Zhiyu Zhang, Yingxin Yang

**Affiliations:** ^1^ General Hospital of Northern Theater Command, China Medical University, Shenyang, China; ^2^ Department of Orthopedics, The Fourth Affiliated Hospital of China Medical University, China Medical University, Shenyang, China; ^3^ Shenyang Traditional Chinese Medicine Hospital, China Medical University, Shenyang, China; ^4^ Department of Anesthesia, The Fourth Affiliated Hospital of China Medical University, China Medical University, Shenyang, China; ^5^ China Medical University, Shenyang, Liaoning, China; ^6^ Department of Oral-maxillofacial Head and Neck, Oral Maxillofacial Surgery, School of Stomatology, China Medical University, Shenyang, China; ^7^ Department of Orthopedics, Shanghai General Hospital, Shanghai Jiao Tong University School of Medicine, Shanghai Bone Tumor Institution, Shanghai, China; ^8^ Department of Neurology, The Fourth Affiliated Hospital of China Medical University, China Medical University, Shenyang, China

**Keywords:** tumor therapy, nanogels, functional groups, controlled sustained release, intelligent response

## Abstract

**Introduction:** Cancer remains a significant health challenge, with chemotherapy being a critical treatment modality. However, traditional chemotherapy faces limitations due to non-specificity and toxicity. Nanogels, as advanced drug carriers, offer potential for targeted and controlled drug release, improving therapeutic efficacy and reducing side effects.

**Methods:** This review summarizes the latest developments in nanogel-based chemotherapy drug delivery systems, focusing on the role of functional groups in drug loading and the design of smart hydrogels with controlled release mechanisms. We discuss the preparation methods of various nanogels based on different functional groups and their application in cancer treatment.

**Results:** Nanogels composed of natural and synthetic polymers, such as chitosan, alginate, and polyacrylic acid, have been developed for chemotherapy drug delivery. Functional groups like carboxyl, disulfide, and hydroxyl groups play crucial roles in drug encapsulation and release. Smart hydrogels have been engineered to respond to tumor microenvironmental cues, such as pH, redox potential, temperature, and external stimuli like light and ultrasound, enabling targeted drug release.

**Discussion:** The use of functional groups in nanogel preparation allows for the creation of multifunctional nanogels with high drug loading capacity, controllable release, and good targeting. These nanogels have shown promising results in preclinical studies, with enhanced antitumor effects and reduced systemic toxicity compared to traditional chemotherapy.

**Conclusion:** The development of smart nanogels with functional group-mediated drug delivery and controlled release strategies represents a promising direction in cancer therapy. These systems offer the potential for improved patient outcomes by enhancing drug targeting and minimizing adverse effects. Further research is needed to optimize nanogel design, evaluate their safety and efficacy in clinical trials, and explore their potential for personalized medicine.

## 1 Introduction

Cancer is one of the major diseases that seriously endanger human health. In 2019, the death toll from cancer-related causes surpassed 10 million, nearly doubling the number from 1990. Moreover, the dense population in Asia has exacerbated the challenges related to cancer. (Lin et al., 2021). Chemotherapy is an important cancer treatment, However, traditional chemotherapy drugs can cause adverse reactions of varying severity, common gastrointestinal reactions, serious cases will appear bone marrow suppression and immunosuppression. Moreover, due to the poor selectivity of traditional chemotherapy drugs, non-specific chemotherapy drugs will kill normal cells in the human body while killing tumor cells to a certain extent. Nano-drug carriers have entered the public’s awareness with the recent rapid advancements in nanotechnology. (Arranja et al., 2017).

Over the past few decades, nanocarriers have been extensively utilized for delivering chemotherapy drugs due to their ability to enhance drug targeting, solubility, controlled release, and reduce toxicity (Hare et al., 2017). A variety of systems have been developed for chemotherapy drug delivery, including hydrogels, microspheres, nanospheres, micelles, and liposomes. Nano-drug carriers can selectively release drugs into the tumor environment, prolonging the blood circulation time and the continuous release of drugs, thereby improving the therapeutic effect of drugs and solving the challenges faced by traditional chemotherapy drugs (Abedi et al., 2021).

Nanogels, as a typical nanocarriers, have attracted extensive attention due to their many advantages. Nanogels are a class of hydrogel nanoparticles with a diameter of 1nm-1 μm. Nanogels have good biocompatibility, high dispersion in aqueous media, and good structure design, which makes them an ideal chemotherapy drug system (Kabanov and Vinogradov, 2009). In addition, compared with other nanocarriers such as liposome-based nanoparticles, the high swelling capacity of nanogels in aqueous media enhances their drug-loading capacity. The encapsulation capacity of self-assembled nanogels, which is crucial for the biological effectiveness of drug molecules and biological macromolecules, is attributed to their ample space for both small drug molecules and large biological macromolecules like proteins, DNA, and peptides. This capacity is achieved through hydrophobic and electrostatic interactions. (Vinogradov, 2007; Neamtu et al., 2017). Compared with rigid nanoparticles, nanogels with flexible and soft structures can penetrate tumor vasculature while maintaining the biological activity of protected therapeutic agents (Choi W. I. et al., 2012). In addition, their flexible nature reduces the possibility of their embedding by macrophages and prolonging their cycle life (Beningo and Wang, 2002). Importantly, compared with traditional carriers such as liposomes, which are less stable, nanogels have higher cellular uptake efficiency than other nanogels, This enhances the *in vivo* bioavailability and safety of chemotherapy drugs. (Hasegawa et al., 2005; Ahmad et al., 2006).

However, most nanogels use traditional gel materials, and the release behavior is uncontrollable, and many cross-linking agents have certain toxicity. Therefore, synthesizing new nanogels and cross-linking agents through chemical modification and constructing multifunctional nanogels with high drug loading, controllable release, and good targeting can further improve their advantages in drug delivery (Mitra et al., 2015). According to different nanogels, the ways of connecting chemotherapy drugs are different, but most are cross-linked through their functional groups. As common functional groups on nanogels, carboxyl group, disulfide bond, hydroxyl group, and amino group play a role in directly connecting drugs or stabilizing nanogels. In addition, the existence of many functional groups is considered the key to the intelligent and controlled release of drugs. The common response strategies include pH-sensitive response strategy, redox response strategy, temperature-sensitive response strategy, and light-sensitive response strategy, etc. Different response strategies can be selected according to the characteristics of various tumors and can yield twice the result with half the effort.

The carrier materials usually used to prepare nanogels are divided into natural nanogels and synthetic nanogels. Natural nanogels, such as gelatin, chitosan, hyaluronic acid, alginate, and heparin, have good biological compatibility, but often have the disadvantages of gel difficulty and poor mechanical ability. At this time, synthetic nanogels such as polyacrylic acid and Pluronic have attracted much attention due to their easy access to materials and strong controllability of performance, but there are shortcomings in the functionality of supporting cells and tissues (Ma et al., 2022). Nanogels can be prepared by the amino group-based cross-linking, “Click” reaction cross-linking, physical cross-linking, photo-crosslinking, polyphase monomer polymerization, and other methods. During the preparation process, the various side chain groups have an impact on different aspects of nanogels, such as drug loading, transport, and controlled release. These aspects are highlighted as key points in this paper. Nanogel is an ideal new drug delivery system, which has broad application prospects in chemotherapy drug transport, protein drug transport, gene and so on.

This article mainly reviews the latest development in the preparation of nanogel chemotherapy drug based on different functional groups used in tumor therapy summarizes the application of these functional groups scenes and improve the strategy, the article describes intelligent controlled release methods at the same time. Finally, The application prospect of related drug-carrying nanogel is also prospected.

## 2 Functional groups in the preparation of nanogels

The functional groups in the nanogels can not only connect directly to chemotherapy drugs, but also cross-linking agents to improve stability. The most common functional groups used for cross-linking drugs are carboxyl groups. In addition, disulfide bonds and hydroxyl groups will also be described one by one Table 1.

**TABLE 1:** List of functional groups used in nanogel.

Functiol group	Drug carrier	Application	Size (nm)	ZETA potential (mV)	Purpose	References
Carboxyl group	CDDP-crosslinked DOX-loaded hyaluronic acid (HA) nanogels (CDDPHANG/DOX)	Load CDDP	57.4 nm-86.4		Adjuvant anticancer and stabilizing agents	Zhang et al. (2018)
Carboxyl group	Cisplatin crosslinked dextran nanoparticles loaded with doxorubicin (Dex-SA-DOX-CDDP)	Load doxorubicin	80		Colorectal cancer, breast cancer	Li et al. (2015)
Carboxyl group	Doxorubicin and cisplatin supported polyacrylate-based stimulus-response polymer nanogels	Load doxorubicin	<100	−28 mV to −17	Multidrug-resistant MCF-7/ADR tumor (Breast cancer)	Wu et al. (2017)
Carboxyl group	Poly [methacrylic acid-co-(poly (ethylene glycol) methyl ether methacrylate)-co-dopamine-co-N,N-bis(acryloyl)cystamine], poly (MAA-co-PEGMA-co-DAA-co-BACy) loaded with doxorubicin and bortezomib (BTZ) (DOX-Loaded BTZ-PMPDA)	Load doxorubicin	300		MCF-7 cells (Breast cancer)	Qiu et al. (2021)
Carboxyl group	Doxorubicin-loaded complex polymeric nanogels of poly (acrylic acid-b-N-isopropylamide-b-acrylic acid/polypyrrole) (D-PPy@PNA)	Load doxorubicin	100	−43.6 ± 1.3	HepG-2 cells (Liver cancer)	Geng et al. (2020)
Carboxyl group	Camptothecin (CPT)-conjugated prodrug (CPTP) micelles	Load CDDP	50		Adjuvant anticancer and stabilizing agents	Li et al. (2019b)
Disulfide bond	Doxorubicin-loaded hyaluronic-based nanogels (HNPs), all-trans retinoic acid (ATRA)/aggregation-induced emission luminogen (AIEgen) fluorophores (TPENH2)-grafted hyaluronic acid (HA) (HA-ss-ATRA/TPENH 2)	Load doxorubicin	585-699		HepG-2 cells (Liver cancer)	Ma et al. (2018)
Disulfide bond	Amphiphilic hyaluronan-SS-poly (ε-caprolactone) diblock copolymers (HA-SS-PCL)	Load doxorubicin	120.8-236.4		HepG-2 cells (Liver cancer)	Yang et al. (2019)
Disulfide bond	Hyaluronic acid (HA) and folic acid (FA) conjugated to form amphiphilic polymer (HA-SS-FA)	Load doxorubicin	100-120	−6.7 to −31.5	HCCLM 3 cells (Liver cancer)	Yang et al. (2018b)
Disulfide bond	Camptothecin (CPT)-conjugated prodrug (CPTP) micelles	Load camptothecin	50		4T1 cells (Breast cancer)	Li et al. (2019b)
Amino group	Chitosan/hyaluronan nanogels to co-load methotrexate (MTX) and 5-aminoleavulinic acid (ALA) (MTX-ALA NGs)	Load methotrexate	141.43 ± 0.47	31.59 ± 0.44	Psoriasis treatment	Wang et al. (2022)
Amino group	Polyacryloyl hydrazide (PAH) capped silver (Ag) or gold (Au) nanogels	Load doxorubicin and camptothecin	122 to 226	−22 to −30	MCF-7 cells (Breast cancer)	Ujjwal et al. (2015)
Amino group	The biotin modified N-vinylcaprolactam (NVCL) was grafted onto Chito san and loaded with doxorubicin (DOX) and Indocyanine green (ICG) nanogels (DOX/ICG@Bio-CS-PNVCL)	Load indocyanine green and doxorubicin onto nanogel	115 ± 6		4T1 cells (Breast cancer)	Zhang et al. (2022)
Borate ester bond	Poly (acrylic acid-co-4-vinylphenylboronic acid) nanohydrogels (P (AA-co-4-VPBA) NG) loaded with doxorubicin and combretastatin-A4 phosphate (CA4P) (DOX-CA4P@NHG)	Load doxorubicin and CA4P	247		MCF-7 cells (Breast cancer)	Yang et al. (2018a)
Borate ester bond	Poly [methacrylic acid-co-(poly (ethylene glycol) methyl ether methacrylate)-co-dopamine-co-N,N-bis(acryloyl)cystamine], poly (MAA-co-PEGMA-co-DAA-co-BACy) loaded with doxorubicin and bortezomib (BTZ) (DOX-Loaded BTZ-PMPDA)	Load bortezomib	300		MCF-7 cells (Breast cancer)	Qiu et al. (2021)

### 2.1 Carboxyl side chain

Polysaccharide (PS) is a glycosidic chain bound by glycosidic bond, which is composed of at least more than 10 monosaccharides. It is an important part of nanogel. Homogeneous and heterogeneous polysaccharides, such as chitosan (CS), alginate (ALG), and hyaluronic acid (HA), are derived from either a single monosaccharide molecule or different monosaccharide molecules (Liu et al., 2008). These polysaccharides, with their functional groups, enable non-covalent interactions. For instance, the hydroxyl group in polysaccharides can participate in hydrogen bonding, while the amine and carboxyl groups can engage in electrostatic interactions and metal coordination (Giammanco et al., 2015). Synthetic nanogels, depending on the polysaccharide type, can utilize electrostatic interaction, hydrophobic interaction, and π-π stacking to incorporate various drugs, thereby aiding drug dissolution and enhancing drug bioavailability (Wang et al., 2021).

Alginate (ALG) is the second major class of ionic hydrogels used to construct biomedical applications. ALG, a long-chain polymer consisting of D-mannuronic acid and guloruronic acid, is predominantly found in the cell wall and intercellular mucins of brown algae (Debele et al., 2016). Due to its low toxicity, affordability, ease of hydrogel preparation, and excellent mechanical flexibility, ALG has been extensively utilized in biomaterials research (Lee and Mooney, 2012). The crucial hydroxyl and carboxyl groups in ALG are capable of interacting with divalent cations like Zn^2+^, Ca^2+^, and Mn^2+^ through noncovalent interactions, leading to ion gelation and nanogel formation (Russo et al., 2007). Additionally, according to Wang et al., doxorubicin has been shown to manifest pH-responsive drug release behavior, favorable compatibility, and anticancer effects through its electrostatic self-assembly into ALG/Ca^2+^ (Bazban-Shotorbani et al., 2016).

**FIGURE 1:**
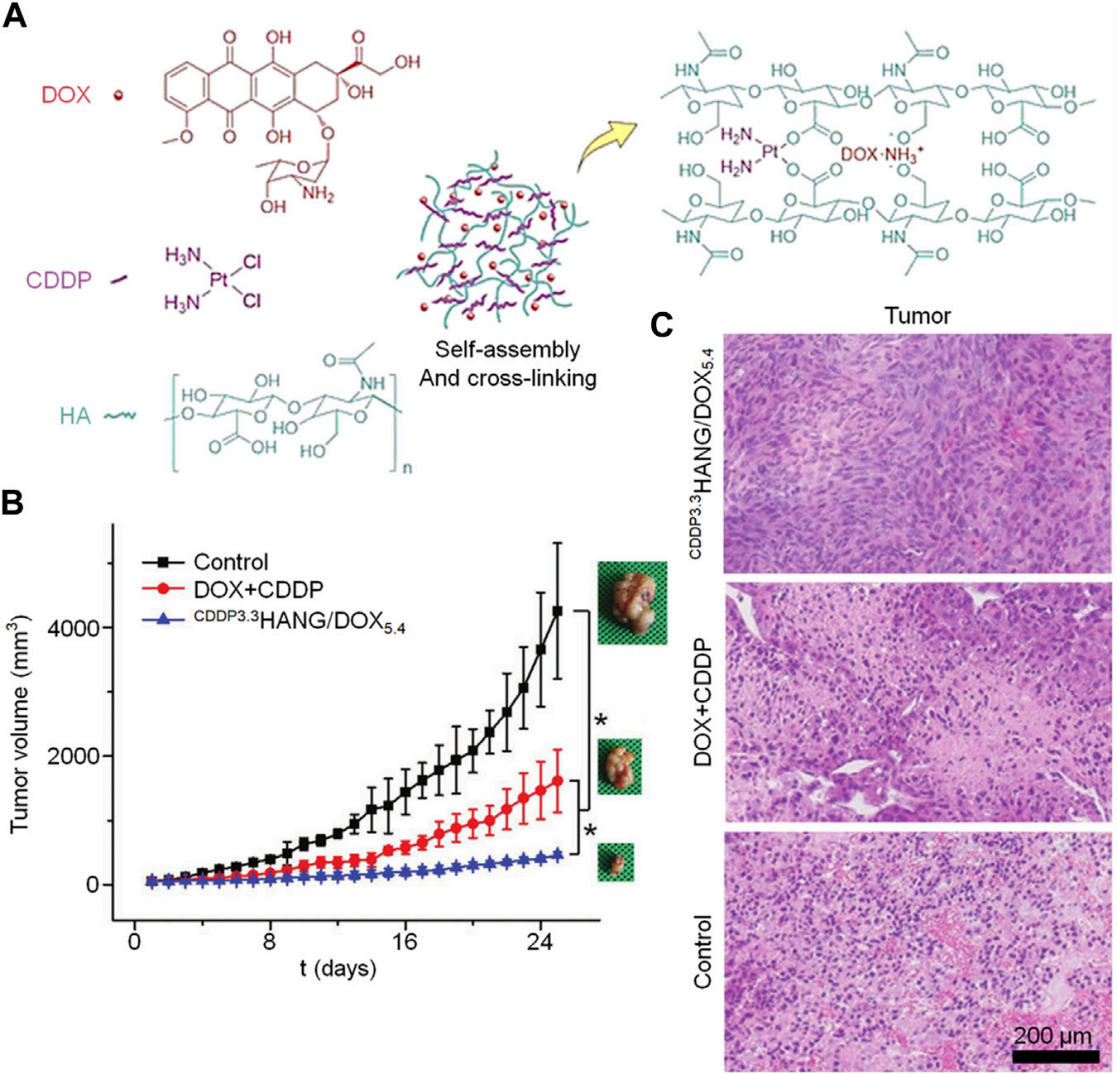
For the treatment of osteosarcoma, a hyaluronic acid-based nanogel is utilized as a carrier for delivering doxorubicin. The following aspects are depicted: **(A)** Schematic illustration of CDDPHANG/DOX preparation, **(B)** K7M2 tumor growth curve, and **(C)** histopathological (H&E) analysis at a magnification of ×200.

CD44-targeted and hyaluronidase-responsive delivery can be achieved with hyaluronidase-based delivery systems. HA can undergo many chemical modifications to obtain different physical and chemical properties, and the commonly used chemical modifications mainly target the following groups on its polymer chain: carboxyl group on glucuronic acid, two hydroxyl groups, and N-acetyl group (Burdick and Prestwich, 2011). Resulting in numerous HA derivatives. The carboxyl group in HA also serves as a non-covalent cross-linking site for Fe3+ and cisplatin. (Wang et al., 2021). Zhang et al. (2018) developed an *in situ* cross-linked nanogel utilizing hyaluronic acid (HA) for treating osteosarcoma. DOX and HA were incorporated into nanoparticles through the electronic interaction of each other’s cations and anions, and CDDP was used as a cross-linking agent to chelate with the side carboxyl group of HA to stabilize the nanogel. Specifically, HA/DOX was obtained by incorporating cationic DOX into nanoparticles through electronic interaction with anion HA (Figure 1A). In addition, to avoid the premature release of CDDP during circulation, CDDP was also used as a cross-linking agent to stabilize the drug-loaded NPs, prolong the circulation time, and reduce the side effects caused by the rapid release of the NPs (Figure 1B). Both the volume change curve of the tumor over time and the histopathology prove that the doxorubicin carrying hyaluronic acid nanogel carrying CDDP has a better therapeutic effect.

Furthermore, HA’s carboxyl group facilitates the controllable esterification reaction, which offers excellent repeatability and control. This reaction is widely used for modification purposes. Highley et al. (2016) aimed to develop an injectable hydrogel with sustained release capability for small molecules. They designed a hydrogel system composed primarily of adamantane-modified and *ß*-cyclodextrin modified HA. By conjugating cyclodextrin with HA, the affinity of HA towards hydrophobic molecules like tryptophan is enhanced. Since many pharmacological small molecules possess circular domains akin to tryptophan’s indole moiety, the increased affinity of HA towards tryptophan facilitates the encapsulation of diverse hydrophobic drug molecules within HA, thereby serving as efficient drug carriers.

In addition to directly linking drugs, the carboxyl group of the carrier gel can also be chelated with cross-linking agents such as cisplatin to improve the stability of drug-loaded nanogels. In their study, it was discovered that carboxyl ligand-functionalized dextran (DEX-SA) efficiently adsorbed adriamycin hydrochloride through electrostatic interactions in an aqueous solution. These dextran molecules self-assembled into polymer micelles of uniform size (DEX-SA-DOX) which were non-cross-linked nanoparticles (NCl-nanoparticles). Consequently, the cross-linking of micelles was utilized to achieve *in situ* synthesis of cross-linked nanoparticles (Cl-nanoparticles, DEX-SA-DOX-CDDP) by exploiting the chelation between platinum (II) antitumor agents and the ionic polymer supports. Notably, the introduction of a small amount of cisplatin as a cross-linker allowed for the *in situ* cross-linking of DOX-loaded polysaccharide nanoparticles, significantly enhancing their surface charge and stability. By comparison to non-cross-linked nanoparticles or free doxorubicin, the cross-linking of polysaccharide nanoparticles with cisplatin brings about an improved anti-tumor efficacy and reduced systemic toxicity. This is achieved through the optimization of biodistribution, controlled drug release, prolonged blood circulation, and enhanced tolerance (Li et al., 2015).

Compared with natural nanogels, synthetic nanogels have better material acquisition and controllable properties, so they are often used as carrier materials for preparing nanogels. Polyacrylic acid (PAA) is formed by the addition polymerization of acrylic monomer polymer materials, has a pH sensitivity, above its pKa (4.75), carboxyl as dissociation state, increases the degree of hydration, Volume expansion (Zhao et al., 2019). Wu et al. (2017) designed a stimulus-responsive polymer nanogel (<100 nm) based on polyacrylic acid as a co-delivery system for doxorubicin and cisplatin. A novel homogeneous stimulation-responsive nanogel was developed using acrylic acid as the monomer. The gel has an abundance of free carboxyl groups, allowing for the creation of complex sites with DOX/HCl and CDDP through strong electrostatic interaction and chelation, respectively. The robust affinity of these interactions enables easy adjustment of the drug loading within the nanogels. In CDDP/doxorubicin combination chemotherapy, the nanogel was utilized, and *in vivo* experiments for anti-tumor efficacy against McF-7/ADR multidrug-resistant tumors showed a remarkable effectiveness with reduced side effects.

The various functional groups and electrostatic interactions used by nanogels to connect drugs are mostly stable in alkaline or neutral environments, however, due to the more acidic tumor microenvironment, the connection is unstable, so pH-sensitive controlled release type nanogels emerged. Qiu et al. (2021) fabricated a ph-responsive polymer nanogel that includes a catechol moiety, a disulfide bond cross-linked structure, and a single carboxyl terminus. This nanogel connects the dangling carboxyl and catechol moieties via classical interactions, while BTZ is linked via boric acid bonds. Given the local acidic environment of the tumor, electrostatic interactions and boric acid bonds that are stable in neutral and alkaline environments can become unstable, followed by the release of BTZ and DOX to achieve therapeutic effects. Polymer nanogels have good stability in physiological environments and can prevent premature leakage of drugs, but the structure of polymer nanogels is destroyed by typical endogenous stimulation. Due to the unstable nature of the nanogels, the release of DOX and BTZ *in vivo* was increased at the same time, which further improved the therapeutic effect of the drugs.

In addition to pH response, photoresponsive nanogels have also attracted much attention. Geng et al. (2020) prepared an *in situ* formed photosensitive injectable drug-loaded hydrogel (D-PPy@PNAs) by a simple method. The main objective of the experiment was to enhance polypyrrole (PPy) by incorporating other monomers, such as polyacrylic acid-b-N-isopropylamide-b-acrylic acid (PNA), in order to improve its photothermal effect, colloidal stability, and photothermal stability. In the initial stage, weak base pyrrole monomers were combined with PNA through acid-base neutralization, leading to the self-assembly of pyrrole-solubilizing PNA micelles. By utilizing the oxidation properties of ammonium sulfate (APS), polyacrylic-B-N-isopropionamid-b-acrylic acid/polypyrrole composite nanogels (PPy@PNA nanogels) with temperature sensitivity were then synthesized through the REDOX polymerization of pyrrole monomers within PNA micelles. This nanogel demonstrates favorable biocompatibility, colloidal stability, high photothermal conversion efficiency, and a responsive sol-gel phase transition behavior triggered by photothermal stimulation. “The π-π superpositions between the backbone of PPy and anthranquinone chemotherapeutic drugs, such as doxorubicin, or the carboxyl groups on PNA are used to link with related drugs through strong ionic bond interactions.” Finally, a photosensitive adriamycin-loaded nanogel (D-PPy@PNA) was formed in the tumor *in situ*, and the controlled release and intelligent response of tumor drugs were achieved.

### 2.2 Disulfide bonds

**FIGURE 2:**
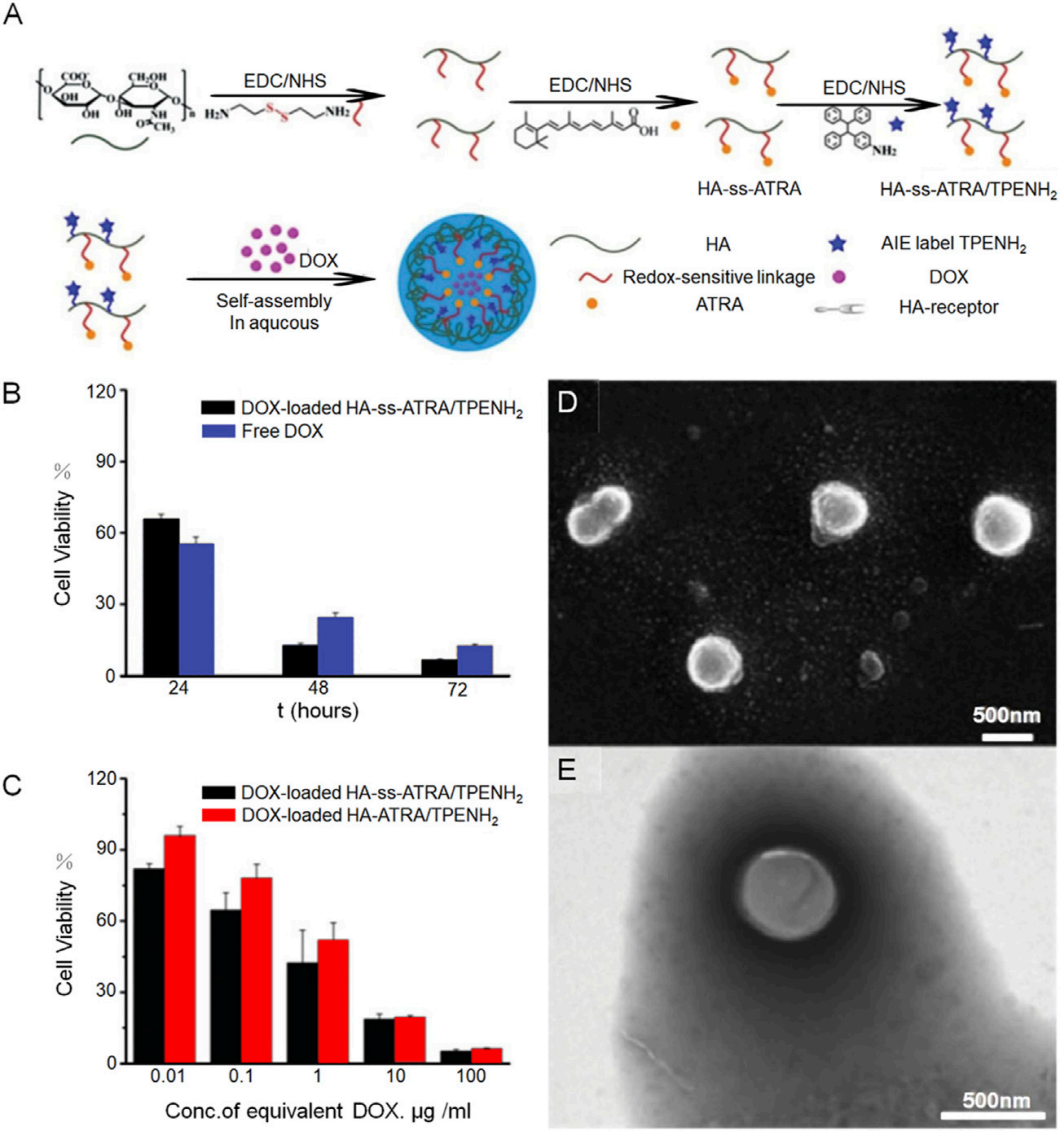
A nanogel incorporating all-trans retinoic acid has shown effective potential in delivering doxorubicin for the treatment of tumors. **(A)** Demonstrates a schematic representation of the process involved in preparing HA-ss-ATRA/TPENH2 conjugates, while **(B)** and **(C)** provide evidence of the robust therapeutic efficacy of DOX-loaded HA-ATRA/TPENH2 from various perspectives. Additionally **(D)** offers representative SEM images, and **(E)** presents TEM images of HA-ss-ATRA/TPENH2 HNPs.

Compared to normal cells, tumor cells generate an abundant amount of free radicals, creating a significant contrast in their internal environment. This distinction can result in DNA damage and cell apoptosis. Furthermore, this unique environment provides an opportunity for drug-loaded nanogels to exhibit sensitivity specifically to REDOX reactions, ensuring drug release occurs primarily within tumor tissues and minimizing potential impact on normal tissues. Disulfide bonds are commonly employed as functional groups in REDOX-responsive drug delivery systems. Notably, cancer cells possess a noticeably higher concentration of glutathione (GSH) in their cytoplasm and nucleus, approximately three times more than that found in normal cells. GSH, acting as a bioreducing agent, effectively cleaves disulfide bonds, leading to the breakdown of disulfide micelles. In the high GSH environment, the breakdown of the mercaptan disulfide bond can easily lead to the destruction of the polymerization network of the nanogel, thus achieving the controlled release of the drug in cancer cells. Disulfide bond modified nanogel has a good effect in promoting drug release. Hyaluronic acid can be used as a targeting agent and hydrophilic agent of HNP, and all-trans retinoic acid can be used as the hydrophobic core of HNP. Ma et al. (Ma et al., 2018) developed an amphipathic conjugate of HA-ss-ATRA by linking hyaluronic acid and all-trans retinoic acid (ATRA) via a disulfide bond. This was followed by ligation of an AIE fluorophore (TPENH 2) containing an amino-terminal and doxorubicin as the main therapeutic drug. The nanogels remain stable in the blood circulation and release the drug by HA receptor-mediated excitation (Figure 2A), SEM and TEM images of HA-ss-ATRA/TPENH2 HNPs were also presented (Figures 2D, E). Through the bar graph of cell viability, we can see the influence of nanogel carrier and disulfide bond on the performance of chemotherapy drugs (Figures 2B, C).

Hyaluronic acid (HA) is a naturally occurring hydrophilic polysaccharide that possesses biodegradable, biocompatible, and non-immunogenic properties. It serves as the primary ligand for the CD44 receptor and is highly expressed on the surfaces of numerous tumor cells. The binding of hyaluronic acid to CD44 is a specific interaction achieved through the interaction of the polysaccharide chain of hyaluronic acid with the ligand-binding domain of CD44, and because it is widely expressed, it is often used to target the delivery of drugs loaded on hyaluronic acid to CD44-overexpressed tumor cells. The synthesis and investigation of a disulfide-bonded amphiphilic copolymer consisting of hyaluronic acid and poly (ε-caprolactone) (HA-ss-PCL) was conducted by Yang et al. (2019). This copolymer was developed as a nanocarrier for the purposes of tumor diagnosis and treatment. The use of dialysis resulted in the formation of therapeutic nanocarriers that encapsulated adriamycin (DOX) and superparamagnetic ferric oxide (SPIO). *In vitro* drug release findings revealed that HA-ss-PCL micelles exhibited the ability to release doxorubicin triggered by a reducing agent, with a release rate of 100% within 12 h under 10 mM glutathione (GSH) treatment, confirming the release potential of doxorubicin from HA-ss-PCL micelles. The results obtained from the MTT assay, cell apoptosis analysis, laser confocal scanning microscope images, and flow cytometry demonstrated that DOX-loaded micelles composed of HA-ss-PCL exhibited a higher level of cellular uptake and cytotoxicity against HepG2 cells when compared to reduction-insensitive HA-PCL micelles. Additionally, the incorporation of SPIO into HA-ss-PCL micelles improved the sensitivity and relaxation of MRI, enabling a more accurate determination of the distribution of drugs within deep tissues.

Similar to HA, folic acid (FA) is a significant targeted molecule that interacts specifically with FA receptors and is also overexpressed in a variety of tumor cells. In a similar direction. In their research, Yang Y. et al. (2018) utilized cysteamine (CYS) containing reduction-sensitive disulfide bonds to crosslink HA and FA. This led to the formation of the amphiphilic polymer (HA-ss-FA) through reduction-sensitive disulfide bonds. Following this, blank micelles of HA-ss-FA and micelles containing doxorubicin (DOX) were prepared and subjected to characterization. The characterization results indicated that HA-ss-FA demonstrated outstanding biocompatibility and did not exhibit any observable cytotoxic effects. Additionally, HA-ss-FA/DOX micelles exhibited an increased release of DOX in a reducing environment. Furthermore, these micelles were observed to enhance the cellular uptake of DOX by CD44 receptor-positive HCCLM3 cells and A549 cells, and to improve the targeted delivery of DOX to tumors in nude mice bearing HCCLM3 tumors.

Borrowing the same principle of REDOX reactive drug-loaded nanomaterials. Li and colleagues (Li Y. et al., 2019) developed micelles of camptothecin (CPT) conjugated prodrug (CPTP), in which camptothecin was attached to PEGylated polyglutamate block copolymers via disulfide bonds to enable redox-triggered drug release. Then, using the carboxyl group (COOH) as the coordination agent, the bistable camptothecin prodrug cross-linked micelles (CPTP/CDDP) with hybrid CDDP-COOH as the cross-linking structure were prepared. The results indicated that CPTP/CDDP demonstrated significant anti-tumor activity, prolonged blood circulation, and controlled the release of CPT.

### 2.3 Amino group

As another commonly used polysaccharide compound, the crosslinking of chitosan nanogels relies largely on amino groups. Given that amino groups often crosslink with hydroxyl groups, we will briefly describe it here. Chitosan is one of the most common self-assembled polysaccharide nanogels used to deliver various chemotherapeutic agents. CS is a linear PS with a positive charge, composed of N-acetyl-D-glucosamine (2-acetamino-2-deoxy-D-glucose) unit connected by *ß*-1,4 bonds. The presence of amino groups makes chitosan soluble in dilute acids (below pH6). Chitosan is the only naturally occurring polysaccharide with positive charge, and its protonated amino group forms polycations, which are easy to form complexes with various synthetic or natural anionic species (Madihally and Matthew, 1999). TPP, a widely used non-covalent cross-linking agent with low toxicity, is a typical example of CS and the most common representative. The electrostatic interaction between the negative charge of TPP and the amine on CS leads to the cross-linking of the two, resulting in the formation of nanogels (NG). CS/TPP NG primarily incorporates anionic drugs through the electrostatic interaction of CS. Furthermore, chitosan-based nanogels can exhibit intelligent controlled drug delivery capabilities. Zhang et al. (2022) reported a biopolymer chitosan nanoparticles (NPs) loaded with indocyanine green (ICG) and doxorubicin (DOX) by self-assembly. Chitosan containing -NH2/-OH groups can be hydrogen-bonded with acetic anhydride and carboxyl-containing N,N′-dicyclohexyl carbodiimide to form CS-RAFT, an intermediate product of drug-carrying nanoparticles, Afterwards, the nanogels were modified with biotin (Bio) and grafted with N-vinyl-caprolactam (NVCL) to enhance temperature sensitivity and targeting capabilities. Upon exposure to near infrared (NIR) light, the indocyanine green (ICG) molecules within the nanoparticles (NPs) convert light to heat, causing a significant phase transition in the NPs and facilitating the release of the encapsulated drugs. Concurrently, the slightly acidic microenvironment at the tumor site leads to increased solubility of the chitosan-based nanogels, accelerating drug release. In both *in vitro* and *in vivo* settings, the NPs demonstrated effective tumor cell eradication, significantly inhibiting tumor growth in xenograft breast cancer mice during relevant animal experiments. The application of amino groups in other compounds will be described later in the text.

In addition to chitosan nanogels, amino groups are also used in other compounds. The amino side chains on the nanogel can stabilize the surface. Ujjwal et al. (2015) reported the synthesis of a simple PH-responsive polyacrylazide (PAH) -capped silver (Ag) or gold (Au) nanogel for anticancer therapy. At room temperature, PAH-Ag or PAH-Au nanoparticles with controllable size and narrow size distribution can be efficiently synthesized by adding AgNO3 or AuCl to an aqueous solution of PAH, eliminating the need for additional reagents. The carbonyl hydrazide side chain functional groups of polycyclic aromatic hydrocarbons act as both reducing and capping agents, facilitating the generation and stabilization of nanoparticles. The -NH_2_ group within the protruding hydrazide section rapidly coats the surface, effectively stabilizing the resulting nanoparticles.

Some nanocarriers are not initially prepared for tumor treatment, but the preparation principle can still give us great inspiration. Wang et al. (2022) has prepared a chitosan/hyaluronic acid nanogel loaded with methotrexate and 5-aminooleic acid for the chemotherapy of psoriasis, namely, MTX-ALA NGs, which is a combination therapy of photodynamic and chemical drugs. CS and HA can be self-assembled to form biomimetic nanogels (CS/HA NGs) by gentle ion gels. CS/HA NGs combine positively charged CS to enhance cellular uptake and CD44 receptor-mediated HA internalization. The nanogels are cross-linked through the utilization of electrostatic interactions, leveraging the attraction between positive and negative charges present in chitosan and hyaluronic acid. The bonding between CS and MTX relies on the hydroxyl group of CS and the amino group of MTX, while the cross-linking involving CS and 5-ALA occurs via electrostatic interaction.

### 2.4 Additional functional groups

In addition to the common carboxyl and disulfide bonds, many other functional groups play a role. For example, hydroxyl-modified micelles are used to bind drugs. Many cancer cells, such as those found in breast and lung cancer, exhibit overexpression of the CD44 receptor on their surface. In contrast, CD44 was expressed at lower levels in normal cells. Additionally, because of its high affinity for the CD44 receptor, hyaluronic acid (HA) can be used as a targeted carrier for delivering drugs to tumor sites. Yu et al. (2020) synthesized a CD44-targeted anticancer drug release system by modifying nanoparticle micelles with hyaluronic acid, combined with doxorubicin and cisplatin, and observed that it significantly enhanced drug release in 4T1(CD44^+^) breast cancer cells under acidic conditions *in vitro* and *in vivo*. Cell uptake and growth inhibition rates were higher than free drugs.

As reversible chemical bonds, boroester bonds can be used to prepare injectable nanogels. In their study, Yang W. J. et al. (2018) devised an injectable hydrogel for the delivery of the hydrophilic drug CA4P. Injectable hydrogels were used to integrate DOX-loaded NG and create a dual delivery system (DOX-CA4P@NHG) through the use of reversible chemical bonds known as boroester bonds. Upon a single injection of DOX-CA4P@NHG, the hydrogel released the antiangiogenic drug CA4P, leading to specific collapse of tumor vasculature. Subsequently, the nanogels dissociated from the hydrogels and released DOX at a slow pace, inducing apoptosis in tumor cells.

The nanogels use functional groups to give gels different properties for loading, transporting, and releasing chemotherapy drugs, which are then administered intravenously. However, Dang et al. (2022) used bioprinting to directly prepare the gel into a gel scaffold that can fill the surgery. The gelatin-sodium alginate and CuO nanoparticle-based 3D-printed hydrogel scaffold (referred to as GEL-SA-CuO) effectively suppressed the recurrence of hepatocellular carcinoma. Hepatocellular carcinoma (HCC), the most common type of liver cancer, accounts for 75%–85% of primary liver malignancies. Although surgical resection is the main treatment for improving overall survival and quality of life, achieving a tumor-free margin, especially for complex anatomical tumors, is technically difficult. This difficulty increases the risk of cancer recurrence and metastasis due to the presence of residual tumor cells. *In vivo*, the implantation of hydrogel scaffolds at the resection site has shown effectiveness in preventing tumor recurrence after primary resection. To create these scaffolds, printable hydrogels were prepared by combining CuO with gel and sodium alginate, resulting in Gel-SA-CuO hydrogel scaffolds with adjustable size and shape through 3D printing. These scaffolds fill excisable defects and provide mechanical support to tissues. Additionally, during the biodegradation of the hydrogel scaffold, CuO nanoparticles in the Gel-SA-CuO hydrogel scaffold are released in a controlled and sustained manner. Importantly, CuO nanoparticles can act as photothermal agents for photothermal therapy and as a source of Cu2+ under acidic conditions, generating intracellular reactive oxygen species (ROS) through Fenton-like reactions. After treatment with GEL-SA-6CuO for 3, 6, 12, and 24 h, the survival rates of mouse hepatoma H22 cells were 70.504%, 54.821%, 36.605%, and 12.802%, respectively. This study may open the door to developing an advanced multifunctional implantable platform for the elimination of postoperative recurrent cancer.

## 3 Controlled release of nanogels

The stimulation-responsive strategy of nanogels is to design nanogels with different structures and properties according to different conditions to perform conformational changes in order to release their encapsulated drugs to kill tumors. Due to their ability to degrade within cellular environments and their adjustable physical properties, biodegradable drugs hold enormous potential in intelligent drug delivery systems. These drugs offer promising applications in a variety of advanced technology-driven drug delivery systems. Compared with non-degradable materials, biodegradable materials have reduced toxicity *in vivo* (Cheng et al., 2013). Moreover, biodegradable nanoparticle carriers (NGs) can be modified with stimulus-responsive groups, enabling them to selectively target specific cells or tissues *in vivo*. These NGs can then undergo spatially triggered bond cleavage, leading to the controlled release of therapeutic agents at specific time intervals, thus maximizing therapeutic efficacy (Abedi et al., 2021). This section mainly introduces the response strategies for the *in vivo* tumor microenvironment such as lower pH, higher REDOX, and higher temperature, and for the external active excitation conditions such as light and ultrasound.

### 3.1 PH sensitive response strategy

The microenvironment of tumor cells exhibits a lower pH (pH 6.5-7.2), which is weakly acidic compared to the pH of normal tissue and blood (pH 7.4). Furthermore, the pH within the endosomes and lysosomes of tumor cells is further decreased (pH 4.0-6.0). (Zou et al., 2014). Therefore, researchers have designed PH-responsive strategies to deliver drugs that are stable at physiological pH, and deliver drugs specifically at tumor sites by sensing pH changes in the tumor microenvironment, such as swelling, surface charge reversal, or chemical bond breakage. This significantly enhances the therapeutic effect of drugs and reduces their adverse reactions. Nanopolymers are often prepared by connecting pH-responsive broken chemical bonds, and commonly used pH-responsive bonds include 2, 3-dimethyl maleic anhydride, benzoimine bond, thiopropionate bond, hydrazone bond, imine bond, protonate bond, keto, limonic anhydride, acetal and cycloacetal (Aslani and Namazi, 2023).

To date, various nanogels responsive to pH have been devised using acid-sensitive bonds like acetals and ketones, as part of their strategies for controlled drug release (Murthy et al., 2002; Murthy et al., 2003; Tang et al., 2011; Zhou et al., 2015). The breakdown of ortho-ester bonds induced by acid is commonly employed in the development of pH-responsive polymers and nanogels for delivering drugs both inside and outside cells. These ortho-ester bonds exhibit stability in neutral conditions, such as blood circulation, but undergo rapid hydrolysis to release drug carriers at low pH levels. Unlike acetals and ketones, the hydrolysis of the ortho-ester bond can exhibit increased sensitivity in mildly acidic conditions and its hydrolysis rate is influenced by the hydrophobicity of the surrounding environment. (Tang et al., 2009; Cheng et al., 2012). Therefore, by optimizing the hydrophilicity around the o-ester bond, the o-ester-based nanocarriers can be excited efficiently under mildly acidic conditions. In addition, ortho ester-based nanocarriers have good biocompatibility and stability in neutral environment (Xu et al., 2014; Wei et al., 2015). Various types of acid-intolerant polymeric drug carriers prepared by ortho ester bonds have shown great potential excellent stability in neutral environments and good drug release performance under mildly acidic conditions.

Li S. et al. (2019) successfully synthesized an acid-responsive nanogel (NG1) by using ortho-ester crosslinker (OEDe) to crosslink CMCS, and they also created a non-sensitive nanogel (NG2) by crosslinking CMCS with a different crosslinker (EGDE). Both nanogels have good stability in the physiological environment. In contrast to NG2, NG1 exhibits Ph-triggered size changes, degradation, and drug release due to hydrolysis of unstable ortho-ester bonds in a mildly acidic environment. The investigations on the uptake and toxicity of NG1/DOX have revealed that cancer cells readily internalize it, leading to the subsequent release of DOX in the cytoplasm. Additionally, NG1/DOX exhibits enhanced accumulation in multicellular tumor spheroids (MCTS) and effectively delivers a higher quantity of DOX to the core regions compared to the non-responsive NG2/DOX, resulting in amplified anti-tumor efficacy.

**FIGURE 3:**
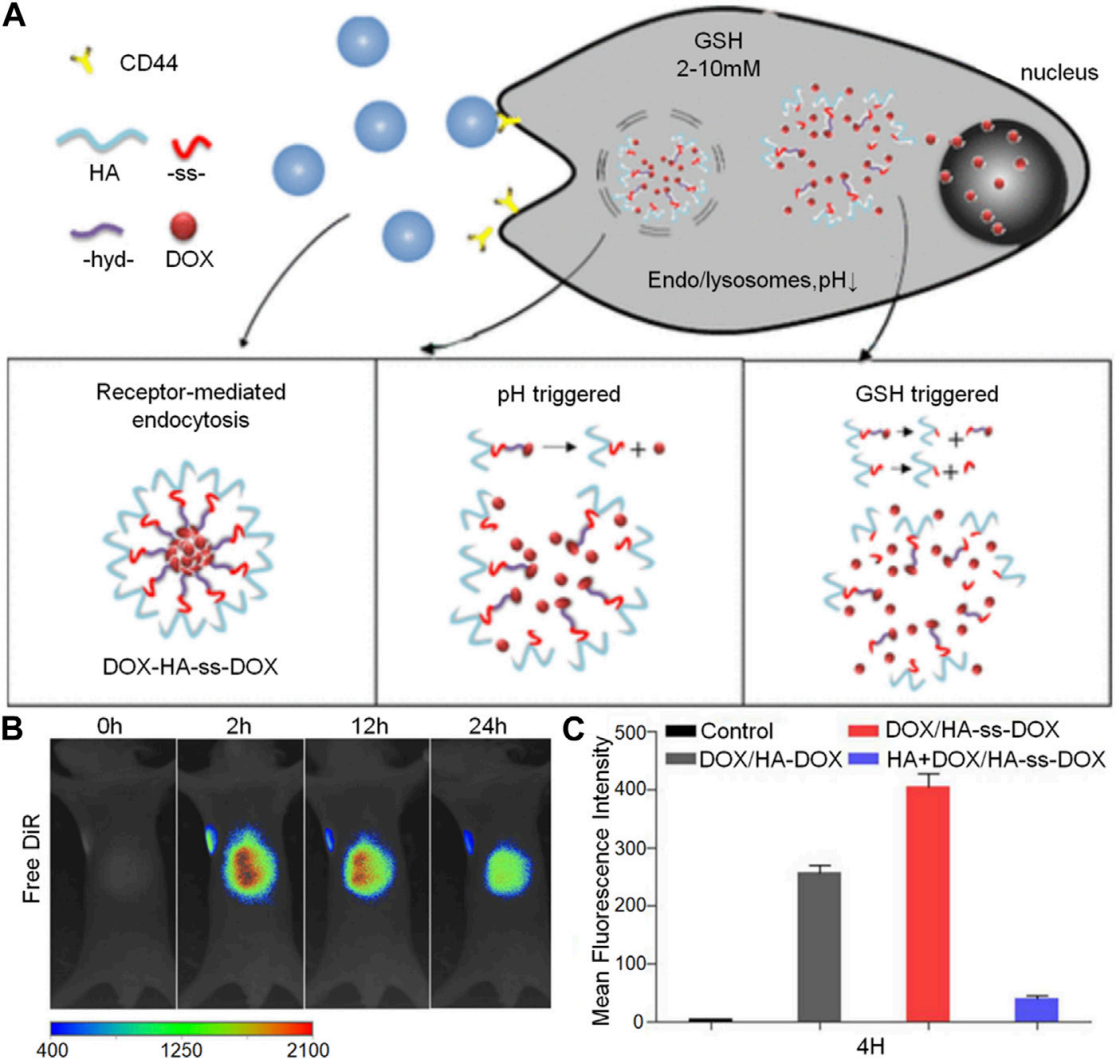
The hyaluronic acid-based nanogel loaded with doxorubicin can release the drug in response to receptor stimulation, changes in pH, and high levels of GSH. The figures show: **(A)** The preparation process for DOX/HA-ss-DOX illustrated schematically, **(B)** *In vivo* imaging of mice with tumors after the administration of free DiR at different time points (2, 12, and 24 h) using the DiR channel, and **(C)** Flow cytometry data from A549 cells, providing quantitative evidence of the remarkable efficacy of DOX/HA-ss-DOX.

In addition to the pH-response strategy based on acetal, keto, and o-ester bonds, the pH-response strategy based on hydrazone bond is also of great interest. Liu et al. (2018) created a new type of nanoparticle system which responds to two different pH levels and acts as a versatile method for treating breast cancer by combining immunotherapy and chemotherapy. They used polyL-histidine (PHIS) to encapsulate the R848 anti-tumor immunomodulator, producing the core of the nanoparticles known as PHIS/R848. By binding doxorubicin to hyaluronic acid using an acid-labile hydrazone bond, they then coated it onto the outer surface of the PHIS/R848 core to form the HA-DOX/PHIS/R848 nanoparticles. The hydrazone bond in HA-DOX breaks at pH 5.5 (lysosomal/inner pH), increasing the release of DOX and enabling its cytotoxic effect. Yin et al. (2018) reported the development of a pH and reductive co-responsive prodrug conjugate, HA-ss-DOX, by attaching doxorubicin onto the hyaluronic acid framework using hydrazone and disulfide bonds. Under normal physiological conditions, the DOX/HA-ss-DOX micelles remained stable and showed selective and rapid release of DOX in acidic pH and/or highly reducing conditions (Figure 3A). Their sensitivity to both acidic and reducing environments led to accelerated release curves of DOX in cells. Evaluation in the A549 cell line and xenograft model revealed that DOX/HA-ss-DOX exhibited the most powerful cytotoxic and apoptosis-inducing effects among all tested groups (Figure 3C). Changes *in vivo* imaging of tumor-bearing mice were observed under excitation at wavelengths below 720 nm and emission at 790 nm for the DiR channel (Figure 3B).

### 3.2 REDOX response strategy

**FIGURE 4:**
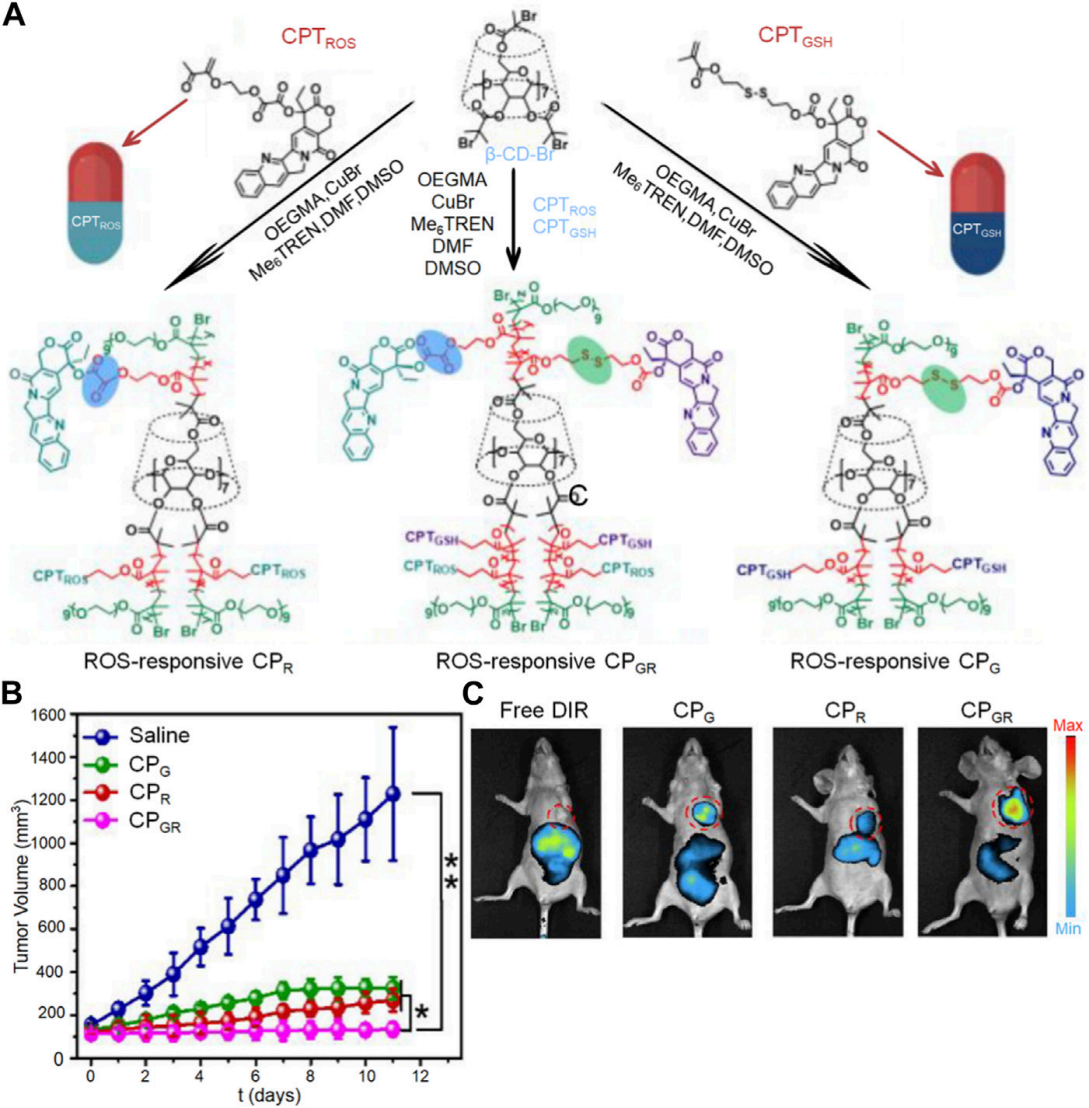
The nanogel loaded with camptothecin is designed to respond to both ROS and GSH for drug release. **(A)** Illustration depicting the preparation process of the dual stimuli-responsive CP polyprodrug and CP micelles, **(B)** Monitoring the changes in tumor volume over time, and **(C)** Studying the *in vivo* distribution of CP micelles.

GSH, a prominent reducing agent in biological processes, is composed of the amino acids glutamate, cysteine, and glycine (Thambi et al., 2016). It is present at significantly higher levels in the cytoplasm of tumors (2–10 mmol/L) compared to its extracellular concentration (2–20 μmol/L). The GSH levels in tumor tissues are at least four times higher than those in normal tissues, as emphasized by Choi K. Y. et al. (2012). The disulfide bond is the most commonly used chemical bond in REDOX response strategy. It is stable *in vivo* and reduced to the sulfur group by a high concentration of GSH after entering the tumor cytoplasm, which changes the conformation of nanocapheres and releases the encapsulated drugs (Gaspar et al., 2015). In their research, Bai et al. (2019) designed a highly sensitive bi-redox prodrug star polymer *ß*-CD-b-P(CPTGSH-co-CPTROS-co-OEGMA) for synergistic chemotherapy, as illustrated in Figure 4A. The tumor microenvironment contains elevated levels of glutathione and reactive oxygen species, which exist in a dynamic equilibrium. This characteristic enables the CPGR micelles to disintegrate upon stimulation, facilitating the controlled and intelligent release of the anticancer drug camptothecin. The changes in tumor volume curves and drug biodistribution in mice, as observed in the images, provide evidence of the superior performance of this dual-stimuli-responsive drug (Figures 4B, C).

Behroozi et al. (2018) have successfully created detachable micelles responsive to redox using the PCL-(SESE-PEG-FA)2 triblock copolymer, which includes redox-sensitive bonds. The diselenide bond, located between hydrophilic and hydrophobic chains, acts as the redox-sensitive bond, leading to the formation of detachable micelles. These micelles can encapsulate hydrophobic anti-cancer drugs (PTX) within their hydrophobic core. The release of the drug is facilitated by the shedding of the micelle shell under specific REDOX conditions.

### 3.3 Temperature responsive gels

Temperature-sensitive hydrogels have been the focus of significant attention over the last 10 years, primarily due to their promising potential for use in biomedical applications. These gels undergo changes in their swelling behavior in response to variations in temperature, consequently enabling the delivery of different drugs. Indeed, the temperature alteration serves as a signal governing the release of drugs. (Hoffman, 2014). Temperature-responsive gels can be utilized in implantable or injectable formats. The primary synthesis mechanism of injectable heat-sensitive hydrogels is thermal gelation, and microgels contribute to *in situ* gel formation above the lower critical solution temperature through thermal gelation. (Askari et al., 2020).

In view of the characteristics of high local temperature in the tumor microenvironment, temperature-sensitive nanogels are selected. Temperature-sensitive gels refer to the preparations that can be delivered in a free-flowing solution state, and can be immediately transformed into gels in response to temperature changes at the administration site after administration (Zhao et al., 2019). It has a high hydrophilic three-dimensional network structure and good biocompatibility. With unique solution-gel transition performance, the drug can be administered in a low-viscosity solution state, which is convenient for administration. After administration, the drug can be immediately transformed into semi-solid or solid gel at the drug site, forming an efficient drug reservoir, improving drug stability, maintaining local continuous drug release, and reducing the number of administration times. The common carrier materials of temperature-sensitive gel consist of natural and synthetic polymers. Natural polymer includes cellulose derivative, chitosan, xylan, etc. Synthetic polymer consists of poloxamme, poly (N-isopropyl acrylamide) PNIPAM *et al.* (Jeong et al., 2002; Klouda, 2015).

**FIGURE 5:**
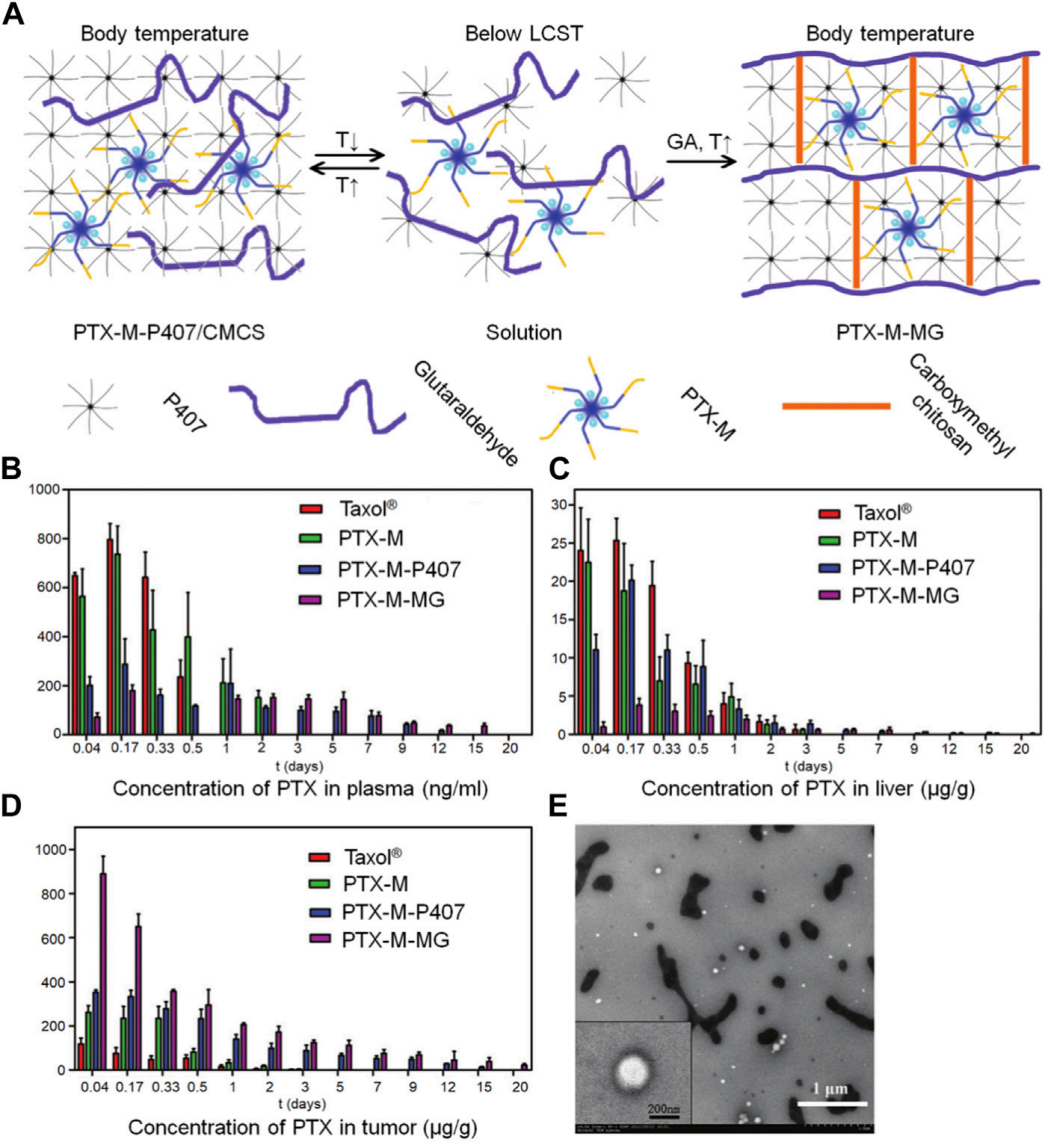
The paclitaxel-loaded chitosan-based nanogel allows for targeted drug delivery by leveraging different tissue temperatures. **(A)** An illustration presents the preparation of the hybrid system based on P407, which includes thermosensitive micelles and hydrogel. **(B)** The distribution of PTX in tissues after intratumoral administration of Taxol^®^, PTX-M, PTX-M-P407, and PTX-M-MG in Heps-bearing mice is depicted in blood **(C)** liver, and **(D)** tumor. Each data point is shown as mean ± SD (*n* = 3). **(E)** TEM images show the release of micelles from PTX-M-MG in a distilled water medium.

As mentioned above, chitosan has good biocompatibility, strong biological adhesion, immune enhancement, anti-tumor, and other biological activities, and is an excellent carrier for the preparation of nanoparticles. Nanoparticles containing anticancer drugs in chitosan have the effect of targeting, slow and controlled release, and can reduce the adverse reactions of anticancer drugs. A combination of chitosan nanoparticles and thermosensitive gels could leverage the strengths of both technologies, providing significant advantages over a single dosage form. Ju et al. (2013) prepared paclitaxel-loaded N-octyl-O-sulfate chitosan micelles (PTX-M) as the carrier, and used different amounts of glutaraldehyde (GA) and carboxymethyl chitosan (CMCS) to cross-link with P407 gel, forming a cross-linked network. This process improved the mechanical strength of the P407 gel and prevented rapid erosion (Figure 5A). In this way, the problems of rapid erosion of local chemotherapy drugs and low drug loading of P407 gel were solved. P407 is a hydrophilic linear triblock polymer that, at a specific concentration and temperature, can undergo micellar and gelation transformation from a solution to a gel. The P407 gel is commonly employed in sustained-release drug systems, particularly for targeted drug delivery, as it can be administered in liquid form and act as a sustained-release reservoir at body temperature. However, the main drawback of P407 gels as sustained-release systems is their rapid erosion in the physiological environment due to dilution by bodily fluids. By incorporating the CMCS-GA cross-linking network, the heat-sensitive P407 gel was transformed into a non-heat-sensitive gel with improved swelling capacity, enhanced mechanical properties, and superior drug release performance. In comparison to unmodified P407 gel (PTX-M-P407) loaded with paclitaxel and PTX-M, PTX-M-MG demonstrated an extended stay at the tumor site for 20 days and a significantly higher tumor inhibition rate of 64.27% (Figures 5B–D). These findings indicate that PTX-M-MG holds great promise as a local hydrophobic drug delivery system, offering the advantages of enhanced efficacy and reduced toxic and side effects.

To achieve local synergistic chemoradiotherapy in hepatocellular carcinoma (HCC), Zheng L. et al. (2019) developed a heat-sensitive complex called Gel-SOR-LUF-SeNPs. Sorafenib (SOR), an important drug for treating unresectable advanced HCC, can cause resistance and serious side effects due to uncontrolled release. In recent years, thermosensitive hydrogels have gained attention as promising drug carriers due to their biodegradability, low toxicity, high drug loading capacity, site-specificity, slow and controlled release. In this study, researchers developed the Gel-SOR-LUF-SeNPs heat-sensitive hydrogel nanosystem as an effective reservoir for continuous localized treatment of hepatocellular carcinoma (HCC) combined with radiotherapy. The results showed a consistent release of SOR over a 15-day period as the hydrogel degraded. The combination of local chemoradiotherapy led to reduced expression of Ki67 and CD34, activation of the caspase-3 signaling pathway, and accelerated apoptosis of HepG2 cells. Importantly, no significant side effects were observed in the tested mice.

In addition to intravenous administration and systemic flow to achieve the targeted location, the gel can also be injected directly into the tumor. Gao et al. (2021) designed an *in situ* drug-loaded injectable thermosensitive hydrogel system to administer norcantharidin nanoparticles (NCTDNPs) and doxorubicin (DOX) simultaneously through the tumor. The NCTD-NPs were prepared using the thin film dispersion method, with the PCEC polymer serving as a support. Subsequently, a heat-sensitive hydrogel based on Pluronic F127 (PF127) was utilized to create a double-loaded gel system by encapsulating NCTD-NPs and DOX. The antitumor activity of this system was evaluated in an H22-bearing mouse model through intratumoral administration. Results: The prepared medication-carrying gel had good heat sensitivity, kept liquid state at room temperature, quickly changed into non-flowing gel at body temperature, and could release drug continuously. *In vitro* studies have shown that, compared with free drugs, potion-loaded gels have significant anti-proliferative activity on HepG2 cells. The *in vivo* experiment evaluating the antitumor effect demonstrated that compared to the other groups, the gel formulation exhibited significant inhibition of tumor growth in H22 tumor mice. Additionally, it also prolonged the survival time of the mice and mitigated side effects. Immunohistochemical staining analysis revealed that the medicine-loaded gel group showed significantly lower expression levels of Ki-67 and CD31 (*p* < 0.05) compared to the other groups. This indicates that the medicine-loaded gel effectively suppressed tumor proliferation and angiogenesis.

Gels can be useful for other purposes than simply delivering drugs, such as reducing the risk of postoperative peritoneal adhesion, as discussed below. Chen et al. (2018) developed a drug delivery strategy to control the release of chemotherapy agents from the intraperitoneal injection of a heat-sensitive poly (n-isopropyl acrylamide) hydrogel (HACPN) with peritoneal anti-adhesion properties. *In vivo* experiments were conducted to observe the chemotherapy and adhesion barrier effects of the anti-cancer drug doxorubicin (DOX) when loaded into hydrogels (HACPN-DOX). The hydrogel’s gradual degradation led to a sustained release following a burst release of DOX. *In vitro* cell culture studies demonstrated DOX’s cytotoxicity against CT-26 mouse colon cancer cells. In an animal model of peritoneal cancer using BALB/c mice injected with CT-26 cells, HACPN-DOX exhibited superior antitumor effects compared to free DOX treatment at an equivalent dose. This was indicated by reductions in tumor weight and volume, improved survival rate, and decreased bioluminescence signal intensity. Furthermore, gross and histological analysis showed that HACPN (or HacPn-DOX) significantly reduced the risk of postoperative peritoneal adhesions resulting from caecum wear in BALB/c mice with neoplastic lateral wall defects.

### 3.4 Light responsive strategy

Photoresponsive hydrogels offer notable advantages in terms of long-range, stable, and immediate delivery compared to other forms of stimulation-responsive hydrogels. (Park et al., 2009; Peng et al., 2010). Photodegraded hydrogels are widely used photoresponsive hydrogels, which can respond to ultraviolet or near-infrared light. By utilizing a light source as a trigger, photoresponsive hydrogels facilitate controlled release of drugs or other substances they encapsulate. This controlled release process occurs due to the absorption of energy from the light, which triggers polymer isomerization and subsequent degradation. (Peng et al., 2012; Yan et al., 2012) The nature of photoresponsive nanogels is mostly temperature-sensitive gels, but the excitation conditions are more demanding. Furthermore, the amount of drug released on demand is directly related to the intensity of the light source. The complex traditional hydrogel preparation process requires strict conditions such as pH adjustments, oxidants, organic solvents, crosslinking catalysts, or UV light. In contrast, thermosensitive hydrogels are easily prepared as they can be injected *in situ* at a specific temperature at the tumor site, and subsequently gel automatically upon temperature change.

Zheng Y. et al. (2019) developed an injectable hydrogel that is sensitive to temperature for safe and effective hyperthermia and chemotherapy of colon cancer *in vivo*. At room temperature, the chitosan (CS) solution was injected into the tumor and then heated to body temperature using *ß*-glycerophosphate acid (β-GP). The strengthening of hydrogen bonds, electrostatic attraction, and hydrophobic interaction between CS and *ß*-GP induces a sol-gel phase transition when the temperature of the CS solution exceeds 37°C. By integrating the photothermal material MoS_2_/bi_2_S_3_-PEG (MBP) nanosheet and the drug molecule doxorubicin (DOX) into the hydrogel, localized photothermal treatment and chemotherapy at the tumor site were achieved. The hydrogel system encapsulated DOX and MBP nanosheets to prevent their entry into the bloodstream and damage to normal tissue cells. Furthermore, the release of DOX within the gel is controllable as the gel’s temperature can be managed through near-infrared laser irradiation. The heat generated during the photothermal conversion process can regulate the rate of drug release, enabling sustained on-demand chemotherapy.

Panda et al. (2021) have developed an innovative mixed drug vector with N-doped mesoporous carbon (NMCS) as the core and PEG-PEI as the shell. Using click chemistry, NMCS was functionalized with a nitrobenzene-based linker. Before the functionalization, gemcitabine was loaded into NMCS via *π*-*π* stacking. The NMCS-linker-PEG-PEI system demonstrates near-infrared and pH responsive properties, enabling the multifunctional drug carrier to release gemcitabine under dual stimuli. The NMCS core converts near-infrared light to ultraviolet light, which is then absorbed by the photosensitive molecular gate, resulting in cleavage and subsequent drug release. Furthermore, NMCS converts near-infrared light to heat, leading to deformation of the polymer shell and initiating the drug release process. The release rate of gemcitabine is anticipated to reach 75% within 24 h under both pH and temperature stimulation. Surprisingly, the gel generated reactive oxygen species (ROS), as confirmed by flow cytometry in FaDu cells, enhancing the cytotoxicity to cancer cells.

Thus, we can see that photosensitized drug-loaded nanogels have many advantages: (1) Activation of these compounds in the near-infrared wavelength range does not result in cytotoxicity even at high concentrations, but upon activation, they exhibit therapeutic effects comparable to those of free drugs. (2) The use of near-infrared light for drug activation presents advantages such as low phototoxicity, increased tissue penetration depth, and reduced background signal, making it highly suitable for a variety of biological applications. (3) Near-infrared thermal imaging can provide valuable information regarding the localization of tumor tissue and real-time feedback on treatment response. (Panda et al., 2021).

More complex multi-sensitive triggering nanogels are also available. Qin et al. (2015) have developed a drug delivery system triggered by near-infrared light, employing amphiphilic chitosan derivatives encapsulated in thermally and pH-sensitive nanogels (CS/PNIPAAm@CNT) with dispersed single-walled carbon nanotubes (CNT). The release of doxorubicin from DOX-loaded CS/PNIPAAm@CNT was faster at 40°C compared to 25°C and at pH 5.0 compared to pH 7.4. Additionally, rapid and repetitive release of doxorubicin occurred with NIR light exposure on DOX-loaded CS/PNIPAAm@CNT. Under NIR irradiation, DOX-loaded CS/PNIPAAm@CNT displayed significantly increased toxicity to HeLa cells, attributed to the NIR-triggered temperature elevation and enhanced release of doxorubicin.

Anugrah et al. (2019) have developed novel hydrogels with near-infrared responsiveness based on the alginate structure, enabling controlled drug release. These hydrogels are rapidly formed by reverse electron demand Diels–Alder click reactions between norbornene-functionalized alginate and tetrazine cross-linkers containing diselenated bonds. Indocyanine green and doxorubicin, both sensitive to near-infrared, were incorporated into the hydrogel matrix during the gelation process. Under physiological conditions, the hydrogels displayed the characteristic of inhibiting drug release, but upon exposure to near-infrared light, rapid release of doxorubicin was triggered. The near-infrared light caused the generation of reactive oxygen species by indocyanine green, initiating the decomposition of diselenated bonds within the hydrogel matrix. This, in turn, induced the gel-sol transition, facilitating the release of encapsulated doxorubicin.

**FIGURE 6:**
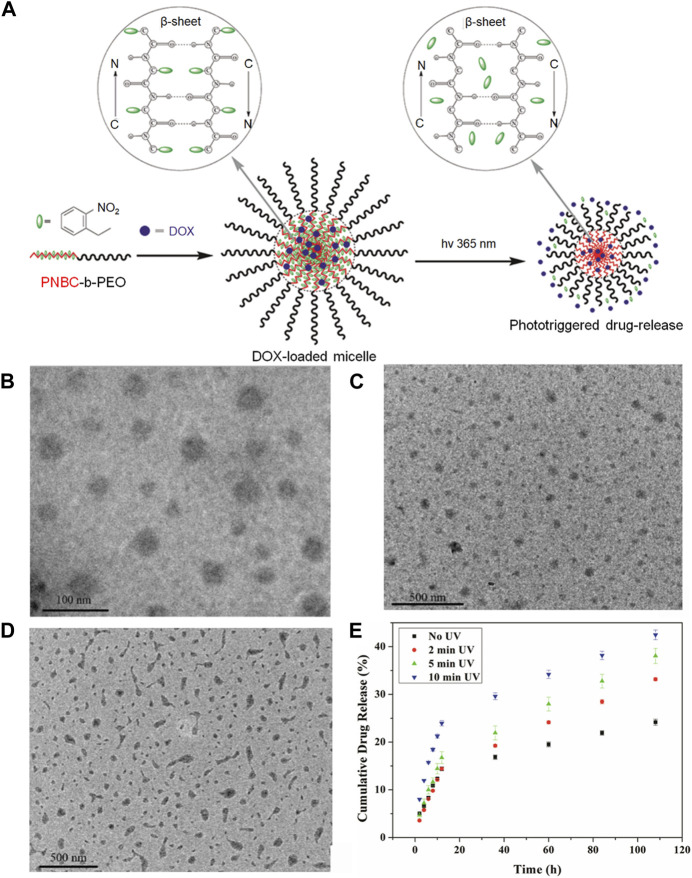
The application of this photoresponsive nanogel loaded with doxorubicin offers numerous innovative possibilities for targeted drug delivery. **(A)** The preparation of the Photoresponsive Poly (S-(o-nitrobenzyl)-L-cysteine)-b-PEO is depicted in the diagram, derived from an L-Cysteine N-Carboxyanhydride Monomer. **(B)** TEM images demonstrate the self-assembly of PNBC-b-PEO nanoparticles obtained from their aqueous solutions: PNBC9-b-PEO. **(C)** The appearance of PNBC9-b-PEO nanoparticles after a 30-min irradiation period is illustrated in the image. **(D)** The depiction portrays drug-loaded nanoparticles of PNBC9-b-PEO. **(E)** The *in vitro* drug release profiles of DOX-loaded PNBC9-b-PEO nanoparticles at 10 mM PBS, pH 7.4, and 37°C, following UV irradiation at 365 nm for various time durations.

In the study by Liu and Dong, (2012), a Photoresponsive Poly (S-(o-nitrobenzyl)-L-cysteine)-b-PEO was developed using an L-Cysteine N-Carboxyanhydride Monomer. They synthesized the poly (o-nitrobenzene methyl)-L-cysteine-B-polyethylene glycol (PNBC-b-PEO) block copolymer through ring-opening polymerization (ROP) of NBC-NCA in a DMF solution at 25°C, as illustrated in Figure 6A. The PNBC-b-PEO copolymer self-assembled into spherical nanoparticles in an aqueous solution, demonstrating photoresponsive self-assembly behavior. Upon irradiation, the nanoparticles decreased in size, and gradual photocracking of the PNBC core of the nanoparticles enabled the release of the anticancer drug doxorubicin by adjusting the duration of light exposure. This study not only offers a convenient strategy for synthesizing photoresponsive polypeptide block copolymers but also for preparing photoresponsive nanomaterials with potential for anticancer therapy. Figure 6E illustrates that longer UV irradiation at 365 nm results in more significant drug release. Additionally, TEM images of PNBC9-b-PEO captured 30 min after light irradiation and drug loading are presented in Figures 6B–D.

### 3.5 Ultrasonic sensitive response strategy

Ultrasound-responsive drug delivery is a new non-invasive, low-cost drug delivery system, and its function is related to ultrasound simulation (Hynynen, 2008; Miller et al., 2008; Qin et al., 2009). Ultrasound presents a type of physical therapy that offers precise spatial and temporal control. Within the ultrasonic reaction chemotherapy system, nanodroplets carrying the drug undergo a transformation into microbubbles when exposed to ultrasonic energy after being injected. (Rapoport et al., 2009; Fabiilli et al., 2010). Under image guidance, ultrasound-responsive nanodroplets serve as an innovative drug delivery system that shows promise in the treatment of various diseases, particularly cancer. These intelligent nanodroplets can be precisely targeted and activated to release therapeutic agents.

At present, most of the ultrasonic response nanogels work together with pH response. For example, Meng et al. (2019) developed an intelligent drug delivery system called O-carboxymethyl chitosan/PFH (O-CS NDs), which demonstrated promising potential in various applications, including cancer treatment. The system comprised of stable nano-droplets (<200 nm) with desirable characteristics such as physical stability, blood compatibility, and low cytotoxicity. These O-CS NDs exhibited strong tumor cell association, excellent serum stability, pH-responsive charge conversion, and efficient ultrasound imaging ability when exposed to low pH environments. The ultrasound-responsive nanodroplets not only facilitated precise drug delivery but also enhanced drug release and absorption under ultrasonic irradiation. The core of the droplets contained perfluorohexane (PFH), which remained in liquid state until exposed to high-pressure ultrasound, triggering vaporization and bubble formation. The doxorubicin-loaded O-carboxymethyl chitosan nanodroplets demonstrated significant cytotoxicity against PC-3 cells when subjected to ultrasonic irradiation. Notably, O-CS exhibited pH-dependent surface charge transfer properties, transitioning from negative charge at near-neutral pH to positive charge in acidic environments. This pH-dependent charge reversal behavior, combined with the ultrasonic response imaging of the nanodroplets, offered several advantages. Firstly, nanoparticles with negatively charged surfaces helped prevent non-specific interactions with blood components, resulting in prolonged blood circulation. Secondly, the cationic properties of the nanoparticles promoted tumor cell interaction within the tumor microenvironment as they circulated. Lastly, the nanodroplets could be easily detected through routine ultrasound imaging techniques.

**FIGURE 7:**
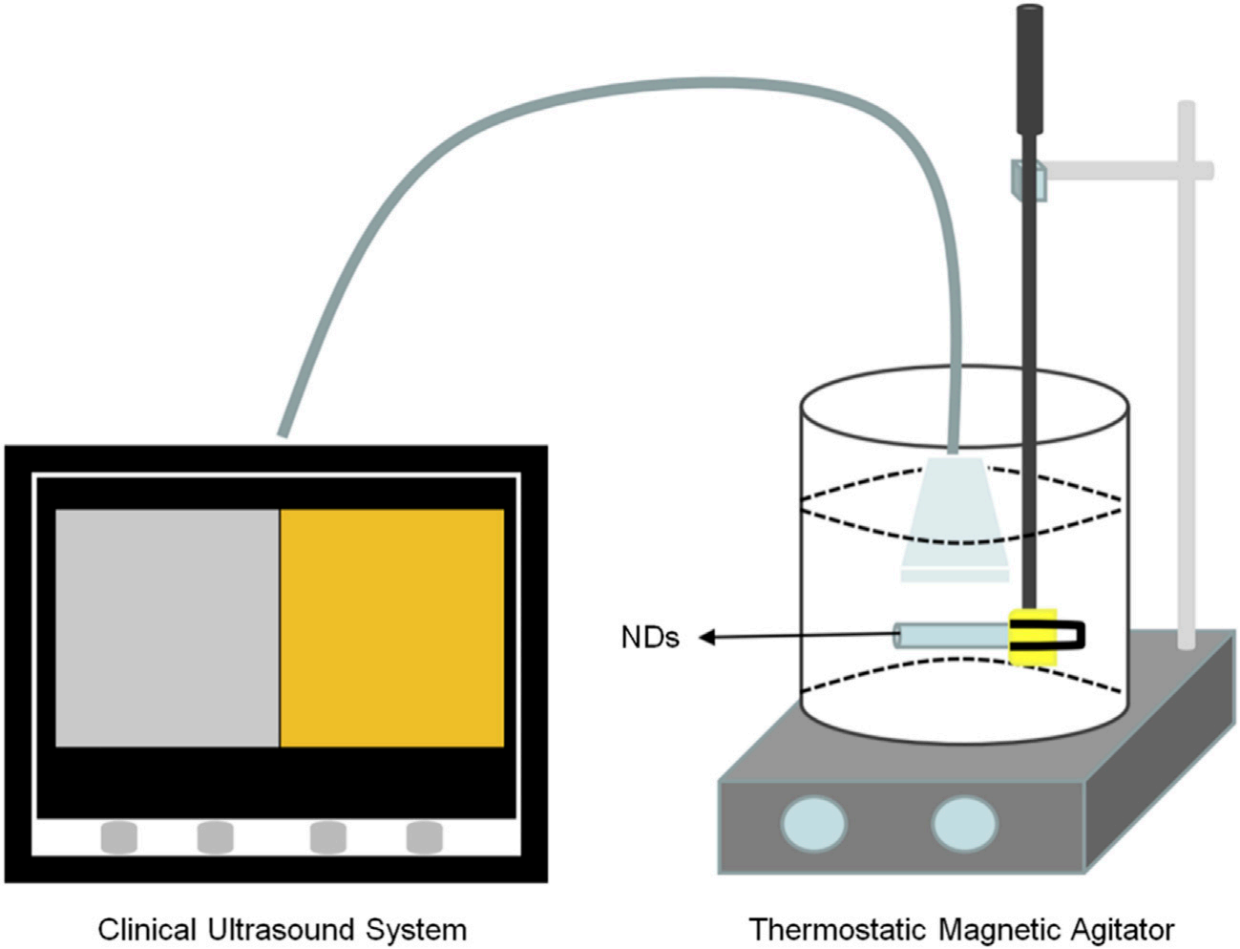
A Setup to monitor the echogenicity of NDs as UCAs for ultrasound imaging.

Shang et al. (2020) developed a novel perfluoropentane-coated nanodroplet consisting of paclitaxel-loaded carboxymethyl chitosan. This nanodroplet exhibited unique responsiveness to both pH and sonographic stimulation, allowing for combined imaging and synergistic radiotherapy (Figure 7A). It has high echogenicity, dosing ability, and radiosensitization ability. Ptx-loaded NDS is a feasible new enhancement method for joint imaging and collaborative radiotherapy.

### 3.6 Other response modes

In addition to the common response modes of nanogels described above, some are commonly used by other nanomaterials, such as ROS response and magnetic response.

**SCHEME 1:**
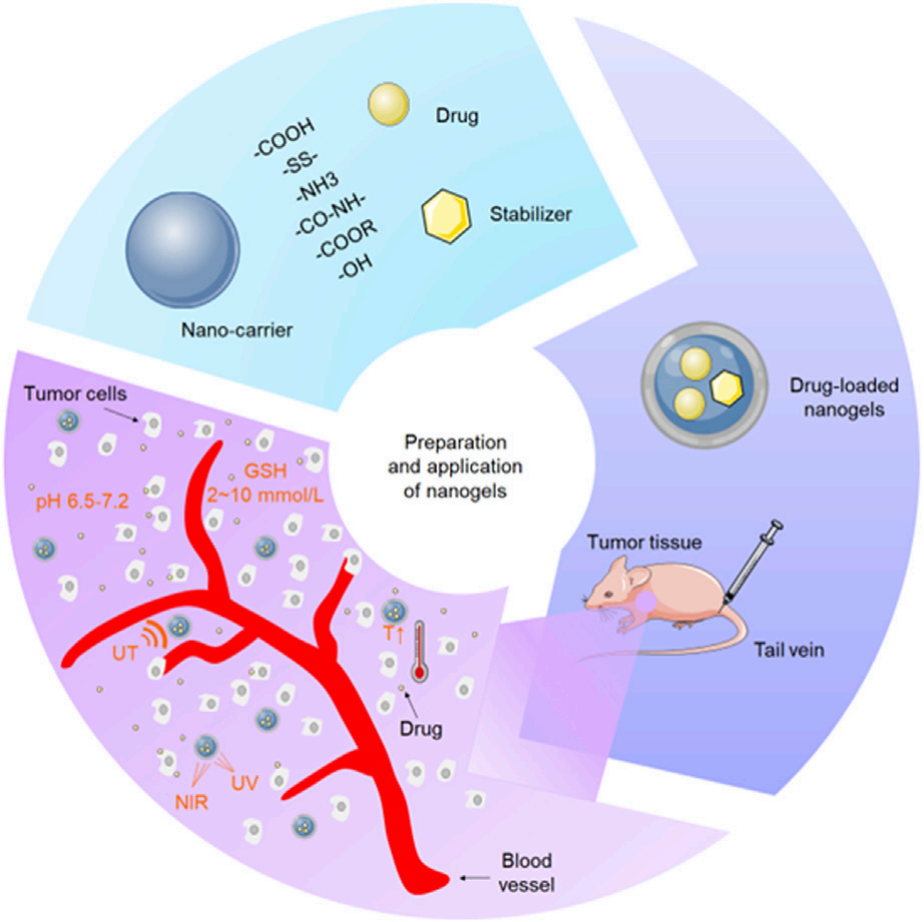
Schematic diagram of preparation and application of nanogels.

The ROS-responsive approach involves the interaction of the nano drug delivery system with ROS in tumor cells, enabling targeted tumor therapy and modulation of the microenvironment’s ROS levels to enhance therapeutic efficacy. ROS, which encompasses hydrogen peroxide (H_2_O_2_), singlet oxygen (1O_2_), superoxide (O_2_), and hydroxyl radical (HO), can undergo interconversion through various chemical reactions (Panieri and Santoro, 2015). An increased risk of cellular DNA mutations, which is closely associated with the progression of several types of cancer cells (Trachootham et al., 2009), is correlated with elevated levels of reactive oxygen species (ROS). Guo et al. (2020) conducted a study where they developed a nanogel (polyprodrug) containing a polymerized platinum (IV) compound that incorporated prodrugs capable of bioreduction and activation under hypoxic conditions, specifically telapazamine (TPZ), to create a synergistic chemotherapy prodrug composition (polyprodrug @TPZ). Analysis using reverse transcriptase polymerase chain reaction (RT-PCR) and a fluorescence probe confirmed the reduction of the platinum (IV) fraction to platinum (II) within the polyprodrug when exposed to the tumor microenvironment. This process notably increases NADPH oxidase (NOXs) expression, leading to accelerated depletion of oxygen (O_2_) and heightened production of reactive oxygen species (ROS). Under highly hypoxic conditions, the activation of TPZ generates cytotoxic free radicals, serving as a secondary antineoplastic agent that synergizes with platinum (II) drugs in chemotherapy. The well-considered design of the nanostructure, comprising the platinum (IV)-based multi-prodrug TPZ complex, provides advantages such as responsive drug release to redox stimuli, effective tumor accumulation, and extended circulation.

Magnetic responsive strategies are widely used in tumor targeting (Tian et al., 2018). Compared with endogenous stimulus response strategies, it can be remotely manipulated by MRI to achieve real-time administration of targeted tissue (An and Zhang, 2017). Magnetic response strategies commonly involve the use of superparamagnetic iron oxide nanoparticles (SPIONs), which typically consist of ferric oxide, ferric tetroxide, and various other ferrites. SPIONs are characterized by small particle size and low toxicity. However, SPIONs are hydrophobic and prone to aggregation, so PEG, PEI, and polyvinyl alcohol should be modified on their surfaces to increase biocompatibility (Santhosh and Ulrih, 2013). In their study, Sun et al. (2018) developed thermal cross-linked superparamagnetic iron oxide nanoparticles (TCL-SPIONs) conjugated with a fibronectin-specific peptide (APTEDB) to create APTEDB-TCL-SPIONs. These nanoparticles were loaded with doxorubicin (DOX) to form DOX@APTEDB-TCL-SPIONs. When the drug was delivered to breast cancer stem cell-like cells guided by magnetic resonance imaging, DOX@APT_EDB_-TCL-SPION was more effective in delivering DOX to the tumor and produced more significant growth inhibition of BCSC tumors than non-targeted DOX TCL- SPIONs.

## 4 Conclusion and prospect

As the basis of nanogel preparation, functional groups produce different effects according to different types. As one of the most common functional groups in nanogel preparation, carboxyl can be directly crosslinking doxorubicin chemotherapy drugs, such as can also be connected, such as cisplatin crosslinking agent to improve the stability of the nanogel, thiol of disulfide bond because of its characteristics of the instability in the acidic environment is often used to make pH sensitive nanogel, acetal, ketal and imine bond had a similar effect, In addition, amide bonds and imine groups formed by dehydration condensation of amino and carboxyl groups also play different roles. In addition to the preparation of nanogels, the controlled and sustained release of nanogels is also an important part of their function. Common strategies include pH response strategy, REDOX response strategy, and temperature sensitive response strategy, which can be selected according to the microenvironment of different tumors.

In summary, the challenges and trends in these fields can be observed. It is evident that the existing methods for preparing nanogels mostly utilize direct nanodispersion methods, which are simple but fail to achieve the desired therapeutic effects, optimal targeting capabilities, and may even give rise to undesired toxic side effects. Bio-3D printing technology can effectively address these issues. Firstly, nanogels, being highly controllable and self-assembling materials, can be accurately printed into designed shapes and structures using bio-3D printing technology, enabling precise control over their physical and chemical properties. Secondly, by utilizing bio-3D printing technology for nanogel fabrication, complex structures and hierarchical spatial distributions can be achieved, resulting in improved biocompatibility and tissue engineering performance. This approach helps researchers better mimic and emulate the microstructure of biomimetic tissues, offering more possibilities for research and application in the biomedical field. Furthermore, bioactive substances such as cells, drugs, or biominerals can be incorporated into nanogels using bio-3D printing technology, enabling targeted release, controlled release rates, and enhanced therapeutic effects. Through the precise printing process, drugs or biocompatible substances can be accurately embedded at specific locations within the nanogels, achieving precise treatment and control. In conclusion, the combination of nanogel preparation with bio-3D printing technology is a promising research direction.
